# Impacts of pine plantations on carbon stocks of páramo sites in southern Ecuador

**DOI:** 10.1186/s13021-021-00168-5

**Published:** 2021-02-09

**Authors:** Carlos Quiroz Dahik, Patricio Crespo, Bernd Stimm, Reinhard Mosandl, Jorge Cueva, Patrick Hildebrandt, Michael Weber

**Affiliations:** 1 Department of Ecology and Ecosystem Management, Technical University of Munich, Munich, Germany; 2 Departamento de Recursos Hídricos y Ciencias Ambientales, Universidad de Cuenca, Cuenca, Ecuador

**Keywords:** Carbon sequestration, Land use change, Carbon pools, Aboveground biomass, Belowground biomass, Soil organic carbon

## Abstract

**Background:**

Since the 1990’s, afforestation programs in the páramo have been implemented to offset carbon emissions through carbon sequestration, mainly using pine plantations. However, several studies have indicated that after the establishment of pine plantations in grasslands, there is an alteration of carbon pools including a decrease of the soil organic carbon (SOC) pool. The aim of this study is to investigate the impact of the establishment of pine plantations on the carbon stocks in different altitudes of the páramo ecosystem of South Ecuador.

**Results:**

At seven locations within an elevational gradient from 2780 to 3760 m a.s.l., we measured and compared carbon stocks of three types of land use: natural grassland, grazed páramo, and *Pinus patula* Schlltdl. & Cham. plantation sites. For a more accurate estimation of pine tree carbon, we developed our own allometric equations. There were significant (p < 0.05) differences between the amounts of carbon stored in the carbon pools aboveground and belowground for the three types of land use. In most of the locations, pine plantations revealed the highest amounts of aboveground and belowground carbon (55.4 and 6.9 tC/ha) followed by natural grassland (23.1 and 2.7 tC/ha) and grazed páramo sites (9.1 and 1.5 tC/ha). Concerning the SOC pools, most of the locations revealed significant lower values of plantations’ SOC in comparison to natural grassland and grazed páramo sites. Higher elevation was associated with lower amounts of pines’ biomass.

**Conclusions:**

Even though plantations store high amounts of carbon, natural páramo grassland can also store substantial amounts above and belowground, without negatively affecting the soils and putting other páramo ecosystem services at risk. Consequently, plans for afforestation in the páramo should be assessed case by case, considering not only the limiting factor of elevation, but also the site quality especially affected by the type of previous land use.

## Background

Afforestation with non-native species in Ecuador started in 1875, when the first species of *Eucalyptus* were introduced with the intention to produce timber, fuel and to restore degraded Andean soils [1–3]. Later, around 1928, seventy species of conifers were introduced including some *Pinus* spp., and after several years of testing, the government implemented afforestation programs with the best adapted species such as *Pinus patula* and *Pinus radiata*. These programs were implemented between the 60’s and 80’s, had total or partial economic assistance, and their main objective was to develop the economy of small producers and rural communities through the production of wood [4–6]. Over the last decades, many pine plantations have been established in order to capture and fix carbon dioxide from the atmosphere through the program PROFAFOR (Programa FACE de Forestación). PROFAFOR is an Ecuadorian company acting as extension of the Forest Absorbing Carbon Dioxide Emissions (FACE) consortium, financed by the Dutch electricity companies to offset their carbon emissions. Since its establishment in 1993, PROFAFOR has signed 152 afforestation contracts with private and community landowners. Until 2003, 22,000 ha of plantations were established in the Andean highlands from which 94% correspond to pine plantations [4, 7]. Most of PROFAFOR ´s plantations are not eligible under the framework of the Clean Development Mechanism (CDM) under the Kyoto Protocol, as their year of planting predates that established in the protocol [7].

While much attention has been focused on the carbon (C) sequestration by growing trees, little attention has been paid to the environmental tradeoffs that are associated with these activities [8]. Most of the programs through afforestation, reforestation, and avoided deforestation have mainly focused on increasing the storage of aboveground biomass (C) [9, 10], without adequately considering soil organic carbon (SOC), even though it can constitute a large fraction of the total C stock [11, 12]. Therefore, there is a growing demand to accurately estimate soil carbon stocks [13] such as páramo soils to evaluate their role as carbon stores. The effects of land use change on soil C are also poorly understood [14], especially in the case of the páramo ecosystem [15, 16].

The páramo, a neotropical high montane ecosystem located between Costa Rica and northern Perú is composed mostly of grasses and shrubs and occurs above the limits of the continuous forest [17–19]. The páramo provides multiple ecosystem services (ES), the most prominent being water supply and regulation, biodiversity conservation, provisioning food for grazing and carbon storage [20–22]. The páramo soils are considered huge carbon stores, because there is a great accumulation of organic matter, due to low temperatures and high humidity that slow down the microbial activity which restricts the decomposition processes [23]. The organic matter, half of which is carbon, generates thick superficial horizons of black or dark tones, classified mostly as Andisols [24]. Recent studies have raised critical views on páramo pine afforestation, considering the potential negative effects on the ES of carbon storage [25, 26].

Research on soil C with afforestation show different outcomes. For example, in a global synthesis Paul et al. [27] found increases and decreases in SOC after afforestation. A subsequent global meta-analysis found that afforestation with pines demonstrated a clear decrease in SOC and nitrogen (N) [28]. The few studies that have been done in the Ecuadorian páramo have found a decrease of SOC [11, 15, 16, 25]. Additionally, in a study in an area of southern Ecuador, Chacón et al. [29] suggested that pines are usually planted on degraded areas or in extensively grazed páramos [25], and for this reason may not be the driver for decreasing SOC. In addition, the effects of land use change on SOC depend on soil properties and environmental conditions, therefore the effects should not be generalized across other regions [25, 30].

With the growing international interest in carbon sequestration, programs for carbon sequestration and conservation are continuously developing [31, 32]. Although currently the establishment of plantations in Ecuadorian páramos is prohibited [33], PROFAFOR contracts on plantations in the páramos allow their landowners to renew the contracts after the harvesting of the plantations [34]. Hence, in order to evaluate the future applicability of these type of programs, it is critical to identify the effects of pine plantations on the carbon stocks of the páramo ecosystem of the region. We have focused our research on pine plantations as they have been the most common land use change for carbon capture promoted in the South region of Ecuador. We especially address the following questions: (i) what are the sizes of carbon stocks and how are they distributed above and below ground in the different types of land use? (ii) what are the effects of different types of land use on the different components of the carbon stock? For this purpose, we have compared three types of land use: natural grassland, which is the dominant vegetation type in the páramo ecosystem studied under natural conditions, *P. patula* plantations, and grazed páramo, which is the most frequent former land use before pine establishment [19].

## Methods

### Study area

**Fig. 1:**
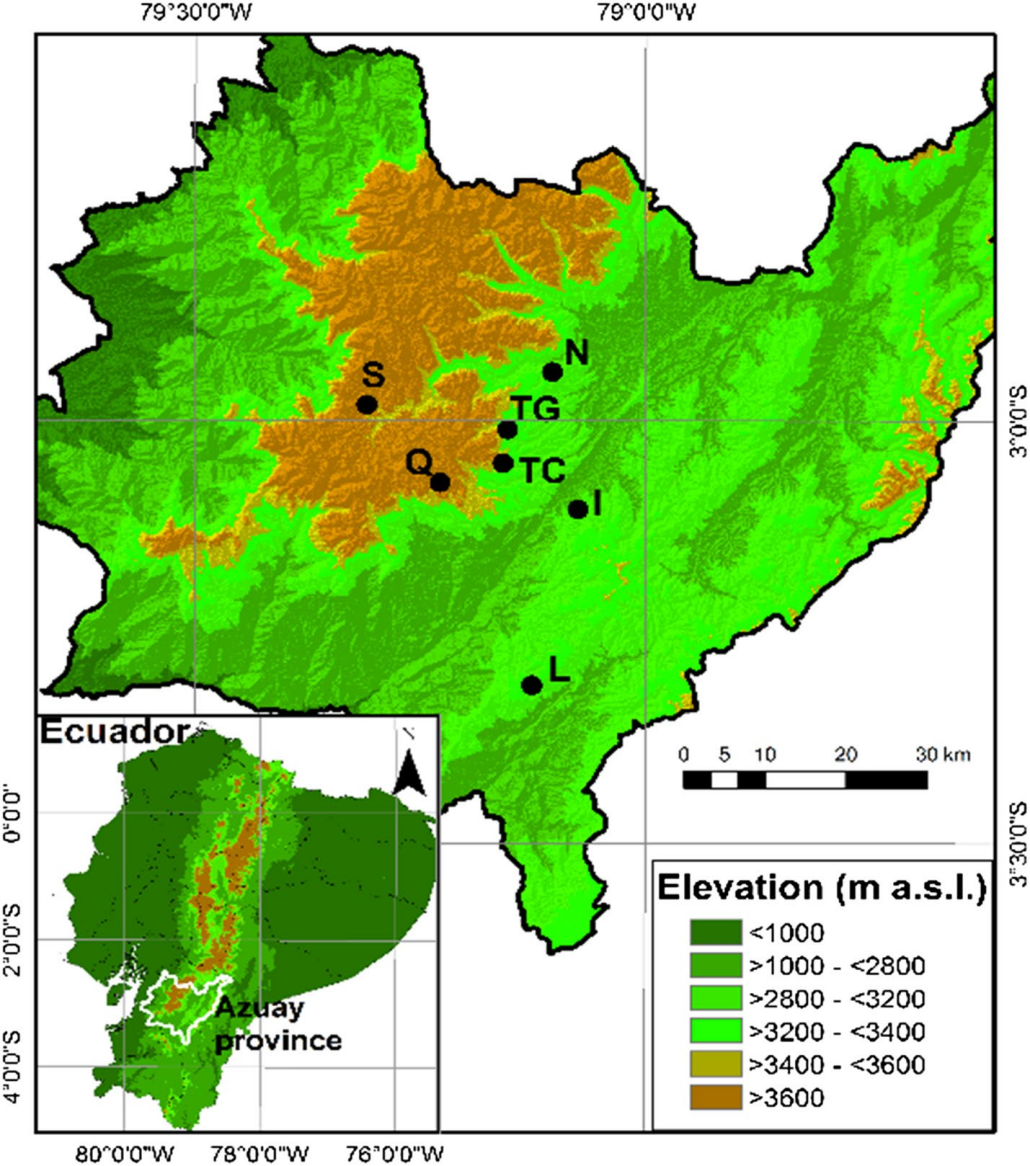
Map of Azuay province showing the seven locations of the study from the lowest to the highest in elevation: Irquis (I), Nero (N), La Paz (L), Tutupali Chico (TC), Tutupali Grande (TG), Quimsacocha (Q) and Soldados (S)

This research took place in the páramo of Azuay province (2° 57′–3° 19′ S, 79° 5′–79° 19′ W), Ecuador (Fig. 1) within an elevational range from 2700 to 3800 m a.s.l. In general the climate in the páramo is wet and humid, and the change in average temperature with elevation is between 0.6 and 0.7 °C for each 100 m of variation in altitude [35]. In this region at 3600 m a.s.l. the average temperature is 8 °C and at 2800 m a.s.l. it is 13.2 °C, while the mean relative humidity is 91% [36, 37]. Rainfall is characterized by frequent low volume events and ranges between 900 and 1600 mm/year [37–39].

The páramo of this region belongs to a landscape that was characteristically shaped by the last glacial period [24, 40]. These soils have been recently (3000 years BP) rejuvenated by a thin layer of fine ash covering the bedrock, most likely due to the activity of the Tungurahua and Sangay volcanos [40]. The volcanic ash is responsible for protecting the humus against decomposition through the formation of organic-mineral complexes [15, 41]. The humid and cold weather and low atmospheric pressure [36] have also favored the store of soil organic matter. This organic matter together with the accumulated volcanic ash are responsible for the formation of black, humid, and acid soils with porous structure, low bulk density, and high water retention capacity [24, 36, 39]. These soils have been classified as non-allophanic Andisols [16, 24]. The study area is located next to the Girón-Paute deflection where the Andes to the south breaks down into smaller mountains whose peaks do not exceed 4.000 m a.s.l., and the treeline is located at a lower elevation [42, 43]. The vegetation is characterized by tussock grass layers, mainly *Calamagrostis* spp. and *Festuca* spp. [44], covering the entire soil surface. Besides these grass species, dwarf shrubs (*Myricaceae*, *Primulaceae*, *Caprifoliaceae*) [44], can be found. The lowest zone of the páramo, located between 2800 and 3500 m a.s.l, is called subpáramo, and it is the transition zone from the Andean forest to the páramo [19]. The vegetation in this zone is composed of elements from the forest below and the grass páramo above. This zone is very difficult to define since the páramo has been affected by human activities since the Holocene (11,000 year BP) [19, 45, 46]. Human activities have changed much of this zone in such a way that now most of the subpáramo zone occur in areas that were probably covered with upper montane forest in the past [47]. The subpáramo communities are predominantly composed of shrubby or woody vegetation that is lacking or limited in the Andean forest below as well as small scattered trees [19, 48]. People living inside or close to the páramo ecosystem tend to have a low average income and depend from their land. Their main livelihood is agriculture, cattle grazing, and in some cases, they have off-farm income [21, 49].

### Experimental setup

To investigate the differences among the C-pools stored in pine plantations and the other land uses, we compared *P. patula* plantation sites (Pi) and adjacent (between 20 m and 2 km) non-plantation sites. We selected two types of non-plantation sites: natural grassland vegetation (NG) which are almost undisturbed sites with the dominant vegetation type in the life zone studied under natural conditions as our control sites, and grazed páramo sites (G), which is the most frequent former land use in the region. All sites were selected so that the main site characteristics (age and management of the plantations, elevation, slope, aspect and soil) were as similar as possible, except for the type of land use. We selected seven locations between 2700 and 3800 m a.s.l., where the three types of land use could be found. In five locations one plantation was selected, and in two locations (Tutupali Grande and Soldados) two plantations were selected.

To measure the characteristics of the plantations, we did a plantation inventory in each one. For the inventory, we used the simple random sampling method. We treated each plantation as single population of *N* units. From the population a sample of *n* non-overlapping fixed-area sampling units (plot) were used. Each plot was randomly chosen. To select each sample plot, we used orthophotos of the plantations as a sampling frame. The orthophotos had a spatial resolution of 0.3 m and were taken in 2010, and proportionated by the “Ministerio de Agricultura, Ganadería, Acuacultura y Pesca”. Each plantation was divided into a grid of square plots, the plots were numbered and randomly selected.

To calculate the sample size, a preliminary sampling was carried out to give an indication of the variability of the population, in which we calculated the coefficient of variation (CV) of pine density per hectare. With this preliminary information we calculated the sample size using a standard method [50]:

1 $$n = \frac{{t^{2} \left( {CV} \right)^{2} N}}{{\left( {N\left( {E\% } \right)^{2} + t^{2} \left( {CV} \right)^{2} } \right)}},$$

where *n* is number of sampling units measured, *t* is the Student’s *t* distribution for desired probability level, *CV* is the coefficient of variation (CV = 100 multiplied by standard deviation and divided by the sample mean), *N* is the total number of sampling units in the population (the plantation), and *E*% is the allowable error as percent of the mean. *t*-value was associated with the 0.95 probability and 4 degrees of freedom, and we set *E*% to a maximum of 20% of the mean based on logistical and cost limitations. In most of the plantations *n* was equal to five. To perform this calculation, we put together the plantations that were at the same elevational range.

Inside each of the nine plantations five plots of 24 × 24 m were established. At each location adjacent to the plantation, we further established six square plots (0.5 × 0.5 m) per land use type (in total 42 plots for G and NG sites along various elevations). Inside each plantation plot we measured the tree height and diameter at breast height (DBH) of all trees to enable the calculation of the biomass of the pines. The coordinates and altitudes were recorded with a GPS for each plot.

### Experimental sites

The páramo has been intervened for thousands of years, a recent study suggests that páramo hunter-gatherers would have actively manipulated the páramo grassland through the use of fire [46]. Until the arrival of the Incas it was used as a ceremony and hunting place. Later, with the development of the Inca culture, it was used for grazing with Andean camelids and for potato cultivation. Then, with the arrival of the Spaniards, it was used for cattle and sheep grazing in addition to cereal cultivation. Currently, the activities that generate the greatest impacts on the páramo are: the expansion of the agricultural frontier, mining, and afforestation with exotic species [51]. For our study we selected three types of land use which correspond to different intensities of human intervention.

#### Natural grassland sites (NG)

Páramo natural grassland sites had characteristics similar to the *páramo in good state of conservation* as described by Hofstede et al. [52] in an evaluation of the conservation status of the Ecuadorian páramo. Natural grassland sites in our study are characterized by the presence of tall tussock grasses, with no recent disturbances such as grazing and burning, a high content of organic matter, and the presence of native fauna. The NG sites in our study were predominantly characterized by tussock grasses, mainly *Calamagrostis* spp*.* and *Festuca* spp. In the lower altitudes the NG sites presented a vegetation typical for the transition zone between the upper Andean forest and the open páramo. These sites were dominated by shrubby or woody vegetation. Thus, the NG sites were not homogenous over the whole elevational range. We did not include small patches of *Polylepis* spp. because they were distant from the pine plantations.

#### Pine plantation sites (Pi)

The plantations were selected based on a forest register provided by the regional forestry department. After contacting 19 plantation landowners, nine of them were selected considering various factors such as plantation size, date of the establishment, average altitude of the plantations, accessibility of the plantation, and the interest of the landowners in cooperating in the research. All selected nine plantations were first rotation *P. patula* plantations with 3 × 3 m spacing located between 2700 and 3800 m a.s.l. The ages of the selected plantations range from 16 to 29 years, with a median age of 19 years. The management of the plantations varied from no management to different intensities of pruning. Thinning was applied in two plantations but in a very limited area which was not significant; so we did not further consider this type of management. These plantations are generally harvested on a 25-year rotation. Six of the plantations were established on extensively grazed páramo (G) and three on páramo natural grassland (NG) (Table 1). Seven plantations were established by the company PROFAFOR Latinoamericana S.A., which is the largest company in Ecuador currently compensating for CO_2_ emissions through afforestation, mostly with *Pinus* species [34].

**Table 1:** Characteristics of the pine plantations (Pi) for each location

Location	Altitud (m a.s.l)	Age (years)	Size (ha)	Slope (%)	Trees per ha	DBH (cm)	Tree height (m)	Management	Type of land use before the establishment of the plantation	Type of vegetation of the undisturbed sites (NG)
Irquis	2780	29	25	46.8	611	26.0	19.2	None	NG	Shrubs, woody veg
Nero	3260	18	30	21.2	694	20.2	11.3	None	NG	Shrubs, woody veg
La Paz	3380	17	46	12.8	850	21.2	9.3	Pruned	G	Tussock grass, shrubbs
Tutupali Chico	3420	16	350	22.4	712	20.8	9.7	Pruned	G	Tussock grass, shrubbs
Tutupali Grande										
Pi1	3460	22	300	26.4	781	17.1	9.0	Pruned	G	Tussock grass
Pi2	3540	20	300	41.1	806	15.8	7.6	None	G	Tussock grass
Quinsacocha	3690	19	123	23.3	573	9.5	4.9	Pruned	G	Tussock grass, shrubbs
Soldados										
Pi1	3760	16	240	20.5	458	12.6	4.8	None	G	Tussock grass
Pi2	3720	19	100	16.8	569	11.2	5.0	Pruned	NG	Tussock grass

*ha* hectare, *DBH* diameter at breast height of the pines, *NG* páramo natural grassland sites

#### Grazed sites (G)

These sites represent the most common former land use in the highland area, grazed páramo grassland [53]. The sites at the higher elevations were dominated by tussock grasses and introduced grasses, *Lolium* sp. and *Dactylis* sp. and in the lower elevations most of the vegetation was dominated by introduced species of *Trifolium* and grasses such as *Pennisetum clandestinum*, *Dactylis* sp. and *Lolium* sp. The sites at the higher locations were managed with extensive cattle grazing and at the lower locations there were higher concentration of animals (two or less animals per ha). There were no signs of recent burning, even though burning of the grasses is a common practice [54]. In most of the sites pre-tilling and reduced tilling activities took place in addition to fertilization (Table 2).

**Table 2:** General description of the grazed páramo sites (G) including their average elevation and slope

Location	Altitud (m a.s.l.)	Slope (%)	Pre-tilling and tilling activities	Type of land use before grazing	Grassland age (years)	Grass	Animal load (ABU/ha)
Irquis	2830	22.5	Preparation through ploughing, liming, and organic fertilization	NG	> 10	*Pennisetum clandestinum*	1
Nero	3200	26.2	Preparation through plowing, organic and inorganic fertilization, and pastures irrigation and rotation	NG	> 10	*Dactylis* sp., *Trifolium* sp*.* and *Lolium* sp*.*	2
La Paz	3320	22.5	Forest logging and burning, solid preparation was made using plowing discs	NG	3	*Dactylis* sp. and *Pennisetum clandestinum*	1
Tutupali Chico	3480	31.5	Tussock grass burn	NG	< 3	*Calamagrostis intermedia*	< 0.2
Tutupali Grande	3470	27.7	Tussock grass burn	NG	< 3	*Calamagrostis intermedia*	< 0.2
Quinsacocha	3600	20.0	Vegetable cover cleaning, soil preparation through plowing and poultry fertilization	NG	5	*Lolium* sp.	0.5
Soldados	3750	14.2	Ground preparation through harrow and adding of vegetal material into the soil	NG	7	*Lolium* sp. and *Dactylis* sp.	0.4

ABU/ha: adult bovine unit per hectare and per year

### Biomass sampling

As no allometric equation to quantify the biomass of *P. patula* in the study region has been developed, and because available equations were developed in different ecosystems [55], we decided to take a small sample of trees to measure their biomass and develop an allometric equation that could be compared and evaluated with the most suitable equations that have been developed. According to Picard et al. [56], even small samples could be valid very locally. Therefore, in each of the nine plantations, one tree with an average DBH and total height for each plantation was selected in the context of the detailed forest inventory realized in each plantation [57].

The selected trees were felled between May and July 2014. From each sample tree, we measured tree height and DBH, then the trees were divided into their components: crown (upper part of the trunk with a diameter < 7 cm), trunk (diameter ≥ 7 cm), branches, needles, and roots. All components were weighed fresh in the field, and a sample of approximately 10% of the weight of each component was taken to the laboratory to be dried. Once the tree was felled, we cut the crown, and then the trunk was cut into 2 m sections. At each trunk section the upper and lower diameter was measured to calculate the volume of each section. At the end of each trunk section a disc was cut, weighed, and taken to the laboratory in order to determine the wood density by dividing the dried mass by the volume (obtained through displacement). All branches of the respective trunk section were weighed and counted, and three representative branches were selected for each trunk section. From the three selected branches, the needles were separated, and then branches and needles were weighed. With these two measurements, we calculated a proportion of weight correspondent for needles and branches. Representative samples from branches and needles were taken to the laboratory. Later the stump was excavated, weighed and a disc sample was taken from it. For the quantification of the biomass of the roots (diameter ≥ 5 mm), two perpendicular axes that crossed in the center axis of the stump were marked and the surface was divided in four quadrants. From two opposite quadrants, one located uphill and the other downhill, all roots were dug out. The roots were classified into three groups, roots with diameters < 7 mm, from 7 to 12 mm, and bigger than 12 mm. Each group was weighed and a sample of 10% of the weight was taken to the laboratory [57]. With the biomass obtained from the nine trees harvested, two equations were fitted, Eq. 2 to estimate aboveground biomass (kg), and Eq. 3 to estimate belowground biomass (kg).

2 $${\text{Ba}} = {\text{Exp}}\left( { - 0.453} \right) \times \left( {DBH^{2} \times h} \right)^{0.649} ,$$

where DBH is the diameter at breast height and h is the height of the pine tree.

3 $$Bb\, = \,{\text{Exp}}\left( { - 0.321} \right) \times \left( {DBH^{2} \times h} \right)^{0.316} ,$$

where DBH is the diameter at breast height and h is the height of the pine tree.

We collected fresh samples from each tree component and dried them at the laboratory. The samples from the crown, trunk, branches, and roots were dried for 72 h at 75 °C to obtain their dry weight, and needles for 48 h at 75 °C. We used the dried samples to calculate the dry/wet ratio, which was later used to extrapolate the dry weight of the entire components. With the biomass obtained from the nine trees, two equations between tree component biomass and the independent variable [squared DBH multiplied by the tree height (D^2^H)] were developed, in the same way as it was done in other studies [55, 58].

The sampling of the ground vegetation was conducted between August and December 2014. For the calculation of its biomass, we aligned and established three subplots (0.5 × 0.5 m) inside each plantation plot, two in the opposite corners and one in the middle of the plot. For the sampling of the aboveground biomass in the non-plantation sites, one plot (0.5 × 0.5 m) was used. For the calculation of the biomass carbon of the ground vegetation, we put together dead wood, litter, and aboveground biomass pools. Dead wood included all non-living woody biomass not contained in the litter either standing or lying on the ground. Aboveground biomass included all living biomass above the soil [59]. All biomass samples were harvested and weighed. We brought the samples from each subplot and plot to the laboratory where we dried them at 75 °C until they reached a constant weight, and they were used to calculate the dry/wet ratio. The biomass of roots was calculated by the collection of one soil sample at three depths: 0–15 cm, 15–30 cm, and 30–45 cm, in each subplot of the plantations and in each plot of the other land uses. We collected the soil samples with soil cores (5 cm in diameter and 5.1 cm length, 100 cm^3^). Soil samples were placed in plastic bags immediately after being taken, and later they were transported to the laboratory. At the laboratory, we sieved the samples with a 2 mm mesh size and all roots were collected, washed, dried (72 h, 75 °C), and weighed using a precision scale. We estimated the biomass carbon content using a standard coefficient of 0.5 [60]. We upscaled dry matter values to t/ha basis.

### Soil sampling

We conducted the soil sampling between August and December 2014. Similar to the root sampling, one soil core was taken in each subplot and in each control plot using metal rings with 100 cm^3^ volume to collect undisturbed soil samples at three depths: 0–15 cm, 15–30 cm, and 30–45 cm. We took the samples to the laboratory of the University of Cuenca in Ecuador, where they were air-dried and passed through a 2 mm sieve to separate fine soil from stones and roots. Fresh fine soil, stones, and roots were weighed. Later the fresh fine soil was oven dried for 72 h at 60 °C to obtain dry fine soil samples. Roots and stones were dried for 72 h at 72 °C. For each dried sample, we calculated the soil bulk density (BD, oven-dry mass of soil per unit of volume) using a standard method:

4 $$BD = \frac{{mass_{fine\;soil} }}{{volume_{sample } {-} volume_{coarse\;fragments} }},$$

where BD is bulk density, mass_fine soil_ is the mass of the fine soil (dried soil that has passed through a 2 mm sieve), volume_sample_ is the total volume of the sample, and volume_coarse fragments_ is the volume of rock fragments and/or roots bigger than 2 mm.

We quantified the volume of the rock fragments and roots by displacement in a water bath. The dry fine soil samples from each depth of the three subplots were mixed for measurement of SOC concentration due to economic reasons. We collected and transferred a portion of 120 g of each soil sample to the Technical University of Munich (Germany), where all samples were ground in a mill (Retsch Mixer Mill MM200) at a vibrational frequency of 25/s for 2 min to obtain a homogeneous sample. These samples were used to measure C concentrations by the dry combustion method using an analyzer Vario EL III. To avoid overestimations of the SOC values, we did not use soil bulk density for its calculations [61]. The quantification of SOC stock was made with the following equations as done by, e.g., Poeplau et al. [62]:

5 $$FSS_{i} = \frac{{mass_{fine\;soil} }}{{volume_{sample} }} \times depth_{i} ,$$

where FSS_i_ is the fine soil stock of the investigated soil layer (t/ha), depth is the depth of the respective soil layer (cm); and:

6 $$SOC_{{stock_{i} }} = SOC_{{con_{fine\;soil} }} \times FSS_{i} ,$$

where ${\text{SOC}}_{{{\text{stock}}_{{\text{i}}} }}$ is the SOC stock of the investigated soil layer (i) (t/ha), and ${\text{SOC}}_{{{\text{con}}_{{{\text{fine}}\;{\text{soil}}}} }}$ is the content of SOC in the fine soil (%), and FSS_i_ is the fine soil stock of the investigated soil layer (t/ha).

### Data analysis

We developed allometric equations between tree component biomass and the independent variable [squared DBH multiplied by the tree height (D^2^H)] using curve fitting with the software SPSS, v. 24.0 [63]. The data were checked for normality with the Kolmogorov–Smirnov test, and for equality of variances with Levene’s test. We used one-way ANOVA when these assumptions were met. Where differences among land uses were significant, Kruskal–Wallis one-way ANOVA on ranks analyses were used to compare means. The significance of the relationships among soil properties was tested using Pearson’s product-moment correlation test (R) and regressions calculated with the function resulting in the highest coefficient determination (r^2^). Except for the development of the allometric equations, the rest of the statistical analyses were done with programming environment R v. 3.5.3 [64] using the *agricolae* package [65], with differences in the p < 0.05 significance level. Mean values for sites or properties were given with the standard deviation in parenthesis.

## Results

**Fig. 2:**
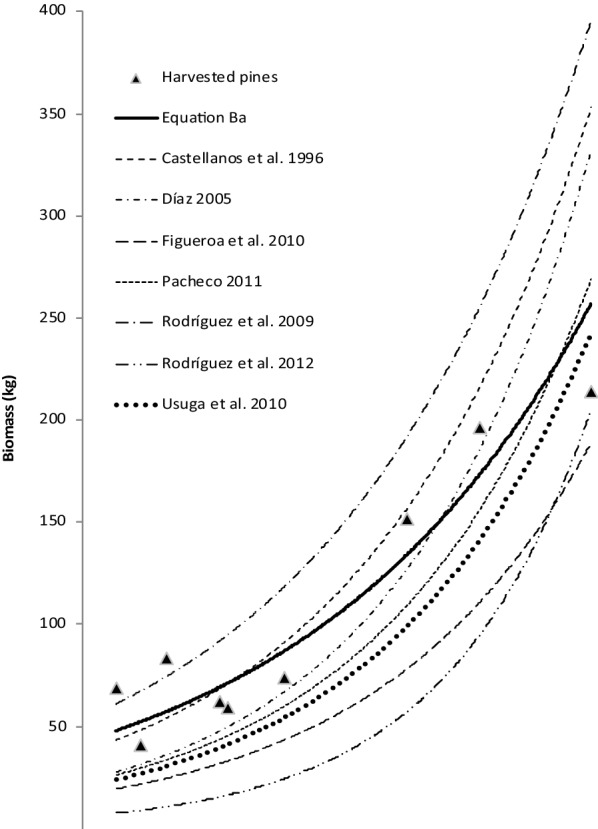
Aboveground biomass estimation curves calculated with the allometric equations developed for *P. patula* including the data calculated with Ba (Eq. 2). The graph includes the biomass of the nine harvested pine trees

Figure 2 shows the results of the aboveground biomass estimation curves derived from the tree biomass analysis in relation to other allometric equations presented in Table 3.

**Table 3:** Information about the studies that have determined allometric equations to estimate the aboveground biomass of *P. patula* trees

Research	Location (state, country)	Elevation (m a.s.l.)	Type of forest	Sampling size (trees)
Castellanos et al. [66]	Puebla, México	2400	Pine forest	27
Díaz Franco [67]	Tlaxcala, México	2875	Pine forest	25
Figueroa et al. [68]	Hidalgo, México	2800	Pine-Oak forest	18
Pacheco [69]	Oaxaca, México	2000	Pine-Oak forest	18
Rodríguez-Laguna et al. [70]	Tamaulipas, México	1800	Pine-Oak forest	111
Rodríguez-Ortiz et al. [71]	Oaxaca, México	2550	Cultivated forest	30
Usuga et al. [72]	Angostura-Manizales, Colombia	2230	Cultivated forest	54

As the values obtained with Eq. 2 were within the range of the results based on the equations found in the literature [66–72], we used our Eqs. 2 and 3 for the further biomass quantifications. Even though most of the equations have been developed in Mexico (Table 3), the relationship between DBH and biomass varied greatly between them. In addition, for the higher values of DBH our Eq. 2 revealed smaller increases in biomass compared to the other equations. This variability between the equations and the risk of overestimating data when using other equations justifies the use of our own equations.

Table 4 provides an overview on the mean carbon stocks in the different compartments and three investigated land uses. In all three land uses SOC represented the dominant part (NG: 91.5%; G: 96.4%; Pi: 80.6%) of the total C stock. The pine plantations revealed the highest mean aboveground and total carbon stock. Nevertheless, the higher aboveground C-stock of the plantations could only partially compensate the substantially lower amount of SOC.

**Table 4:** Mean values of carbon in tons per hectare followed by the standard deviation for each land use

Land use	Aboveground C		Total AG carbon	Belowground C		Total BG carbon	Total SOC	Total carbon stock
	Ground vegetation (litter + herbs + shrubs)	Pines (trunk, branches, leaves)		Roots	Pine roots			
NG	23.1 (17.1)	–	23.1 (17.1)	2.7 (1.6)	–	2.7 (1.6)	275.6 (75.9)	301.3 (76.3)
G	9.1 (5.3)	–	9.1 (5.3)	1.5 (1.3)	–	1.5 (1.3)	282.6 (91.3)	293.2 (91.8)
Pi	14.5 (7.4)	40.9 (27.9)	55.4 (26.8)	3.9 (3.1)	3 (1.3)	6.9 (2.6)	258.0 (78.8)	320.2 (74.0)

*AG* aboveground, *BG* belowground, *SOC* soil organic carbon (0–45 cm)

### The differences in carbon pools among the locations

At all localities, except Soldados, the total aboveground C pools were significantly highest in the plantation sites (Pi) compared to the other types of land use (Table 5). Furthermore, the highest values were recorded in the locations with the lowest altitude: Irquis (106.2 tC/ha), Nero (68.5 tC/ha), and La Paz (68.6 tC/ha). In Soldados, the highest location, the amounts of aboveground carbon were similar between NG (38.2) and Pi1 (35.2) and Pi2 (35.8).

**Table 5:** Mean carbon values of the components of each carbon pool (± standard deviation) [aboveground (AG), belowground (BG) and SOC], for each land use within each location

Location (m a.s.l.)	Land use	Aboveground C (t/ha)			Belowground C (t/ha)			SOC (t/ha)				Carbon stock (AG + BG + SOC)
		(hebs + shrubs + litter + dead wood)	Pines	Total AG	Roots	Pine roots	Total BG	0–15 cm	15–30 cm	30–45 cm	Total SOC	
Irquis (2800)	NG	15.0 (3.8)^a^		15.0 (3.8)^b^	1.2		1.2 (0.4)^b^	85.8 (21.1)^a^	72.8 (17.0)^a^	61.1 (14.9)^a^	219.7 (50.7)^a^	235.9 (48.1)^a^
	G	9.0 (2.7)^b^		9.0 (2.7)^c^	0.6		0.6 (0.3)^c^	67.5 (11.4)^a^	58.1 (11.7)^a^	46.0 (12.8)^a^	171.5 (32.5)^a^	181.1 (33.6)^b^
	Pi	14.5 (5.7)^ab^	91.7	106.2 (14.8)^a^	2.7	4.4	7.1 (1.1)^a^	46.4 (12.3)^b^	39.4 (6.3)^b^	32.8 (3.4)^b^	118.6 (21.4)^b^	231.9 (30.7)^a^
Nero (3230)	NG	21.3 (12.6)^a^		21.3 (12.6)^b^	4.4		4.4 (1.7)^b^	109.8 (23.0)^a^	105.6 (15.1)^a^	94.0 (17.8)^a^	309.4 (46.4)^a^	335.1 (52.9)^a^
	G	18.3 (5.5)^a^		18.3 (5.5)^b^	1.4		1.4 (0.3)^c^	142.9 (53.1)^a^	114.8 (30.0)^a^	106.0 (15.6)^a^	363.7 (85.1)^a^	383.4 (81.8)^a^
	Pi	16.9 (7.4)^a^	51.6	68.5 (15.6)^a^	2.6	3.5	6.1 (1.2)^a^	106.9 (19.8)^a^	105.6 (12.3)^a^	105.4 (23.1)^a^	317.9 (45.7)^a^	392.5 (52.1)^a^
La Paz (3340)	NG	25.1 (18.4)^a^		25.1 (18.4)^b^	2.9		2.9 (1.4)^b^	101.2 (18.0)^a^	95.8 (15.2)^a^	77.4 (10.9)^a^	274.5 (42.4)^a^	302.5 (42.8)^a^
	G	6.3 (1.4)^b^		6.3 (1.4)^c^	1.5		1.5 (0.3)^c^	94.9 (26.4)^a^	89.1 (24.1)^a^	84.0 (31.9)^a^	267.9 (81.5)^ab^	275.7 (80.6)^a^
	Pi	13.9 (2.4)^a^	54.7	68.6 (22.0)^a^	2.7	4.0	6.7 (1.6)^a^	77.8 (18.4)^a^	81.3 (14.3)^a^	65.5 (7.0)^a^	224.6 (28.9)^b^	299.9 (35.1)^a^
Tutupali Chico (3450)	NG	28.0 (17.2)^a^		28.0 (17.2)^b^	3.1		3.1 (1.5)^b^	135.2 (15.0)^a^	124.7 (13.2)^a^	113.3 (16.0)^a^	373.2 (24.5)^a^	404.3 (33.7)^a^
	G	8.5 (4.5)^b^		8.5 (4.5)^c^	1.4		1.4 (0.3)^c^	125.6 (14.2)^a^	124.6 (7.8)^a^	103.8 (7.1)^a^	354.0 (24.2)^a^	363.9 (22.6)^b^
	Pi	9.1 (1.7)^b^	50.0	59.1 (19.0)^a^	4.5	3.5	8.0 (1.6)^a^	123.4 (20.5)^a^	102.6 (10.6)^b^	88.7 (13.5)^b^	314.7 (27.5)^b^	381.8 (32.9)^ab^
Tutupali Grande (3480)	NG	18.8 (15.2)^a^		18.8 (15.2)^b^	2.3		2.3 (0.5)^b^	99.8 (18.0)^a^	79.1 (27.0)^a^	78.6 (25.3)^a^	257.5 (49.5)^a^	278.6 (54.4)^a^
	G	9.5 (4.8)^ab^		9.5 (4.8)^b^	1.2		1.2 (1.3)^b^	95.0 (52.9)^a^	96.8 (8.7)^a^	72.7 (38.0)^a^	264.5 (92.2)^a^	275.2 (92.4)^a^
	Pi1	9.0 (4.1)^ab^	46.2	55.2 (12.9)^a^	1.9	3.5	5.4 (0.3)^a^	117.5 (46.6)^a^	101.0 (39.9)^a^	80.4 (33.6)^a^	299.0 (119.2)^a^	359.6 (120.4)^a^
	Pi2	6.0 (2.6)^b^	34.2	40.2 (12.9)^a^	1.9	3.1	5.0 (1.3)^a^	118.9 (29.3)^a^	113.8 (23.8)^a^	72.0 (5.7)^a^	304.7 (51.8)^a^	349.9 (49.9)^a^
Quimsacocha (3640)	NG	15.2 (6.6)^a^		15.2 (6.6)^b^	1.8		1.8 (0.3)^b^	114.6 (18.8)^a^	110.5 (19.3)^a^	95.6 (22.2)^a^	320.7 (58.7)^a^	337.7 (55.1)^a^
	G	4.7 (1.2)^b^		4.7 (1.2)^c^	0.4		0.4 (0.4)^c^	130.9 (14.3)^a^	114.2 (36.3)^a^	91.9 (24.9)^a^	337.0 (52.9)^a^	342.1 (53.5)^a^
	Pi	14.8 (2.6)^a^	14.8	29.6 (15.4)^a^	5.5	1.8	7.3 (2.9)^a^	93.3 (12.2)^b^	109.0 (25.3)^a^	89.4 (23.1)^a^	291.7 (38.4)^a^	328.6 (49.8)^a^
Soldados (3720)	NG	38.2 (28.5)^a^		38.2 (28.5)^a^	3.0		3.0 (2.3)^b^	88.0 (28.7)^b^	70.0 (21.6)^a^	16.1 (10.1)^a^	174.0 (50.0)^b^	215.2 (60.9)^a^
	G	7.5 (1.2)^b^		7.5 (1.2)^b^	3.8		3.8 (0.4)^b^	115.2 (14.3)^ab^	90.4 (36.3)^a^	14.1 (24.9)^a^	219.6 (52.9)^ab^	230.9 (53.5)^a^
	Pi1	22.6 (10.4)^a^	12.6	35.2 (9.9)^a^	4.5	1.6	6.1 (0.6)^a^	120.4 (7.5)^a^	94.5 (20.7)^a^	13.1 (4.1)^a^	228.0 (25.1)^a^	269.3 (29.9)^a^
	Pi2	23.3 (4.5)^a^	12.5	35.8 (11.5)^a^	8.5	1.4	9.9 (4.6)^a^	119.0 (22.9)^a^	90.5 (27.8)^a^	13.3 (2.8)^a^	222.7 (44.6)^ab^	268.4 (51.2)^a^

*NG* natural grassland, *G* grazed páramo, *Pi* pine plantation

Different letters are significantly different from each other (p < 0.05)

The carbon in the ground vegetation of the plantations is linked with tree density. The less dense plantations such as the ones of Soldados, Quimsacocha, Irquis and Nero (513, 573, 611 and 694 trees per ha, respectively) had the highest amounts (Soldados 22.6/23.3, Quimsacocha 14.8, Irquis 14.5, and Nero 16.9 tC/ha). In contrast, the denser plantations (Tutupali Chico and Tutupali Grande with 712 and 793 tress per ha, respectively) registered the lowest amounts of understory carbon (9.1 and 9.0/6.0 tC/ha, respectively). At all locations the aboveground carbon of the grazed sites (G) was lower than that of the NG sites, at five locations on a significant level, and also the variation among the G sites was much lower.

Regarding the total belowground C pool, there was a clear trend: C pools were highest in all Pi locations, followed by NG, independent from the elevation.

Concerning total SOC stocks (0–45 cm deep), the situation varied among the land uses. At three locations (Irquis, La Paz, Tutupali Chico) the Pi sites had significantly lower SOC pools than the NG and G sites, while at Soldados one plantation (Pi1) had a significant higher pool. At the other locations the SOC pools did not show significant differences. Regarding the share of the deepest tier (30–45 cm) in the total SOC stock it is striking that at the highest location Soldados, it contributed only between 5.7 and 9.3% to the total SOC, while at the remaining locations it was between 23.6 and 33.2%

Despite the differences among the compartments of the several sites regarding the total carbon stock, only the locations of Irquis and Tutupali Chico registered significant differences between the land uses (Table 5).

Concerning the influence of the elevation on the biomass of the pine trees Table 5 shows that above- and below-ground biomass of the pine trees decreased clearly with increasing elevation.

For the three types of land use, the values of SOC got lower with increasing depth (Table 5).

## Discussion

The results of this study provide estimates of carbon stocks of the southern Ecuadorian páramo ecosystem under three types of land use: natural grassland (NG), grazed páramo (G), and pine plantations (Pi). With mean total carbon stocks (above- + below-ground + SOC in the top 45 cm) between 179.3 and 404.3 tC/ha (Table 5), the study confirms the high capacity of the páramo ecosystem as a carbon stock [10, 73]. The most important C-pool of the páramo in all land uses is the soil: in NG an average of 91.5% of the carbon stocks corresponded to SOC, in G 96.4%, and in Pi 80.6%, respectively.

### Comparison of carbon pools among land use types

#### Aboveground carbon

The most noticeable difference in C stock among the land use types in our study occurred in the above- and below-ground biomass carbon pool. Similar to other studies in the Ecuadorian Andes [11, 74], we registered the highest amount of aboveground C in the plantations (Pi). Other studies obtained similar results with native trees [19, 75, 76]. In relation to the different elevation ranges, pines progressively decreased in size with the increase of elevation. The carbon values of the pine trees that we recorded at our two highest elevations (12.5 to 14.8 tC/ha) are similar to those (14 tC/ha) reported in a study conducted in a close area of the páramo at a similar elevation (3800 m a.s.l.) with *Pinus radiata* D. Don [77]. At the lowest elevation (2800 m a.s.l.) the stock was considerably higher (91.7 tC/ha) and compatible with those obtained by Bremer [74] (99–122 tC/ha). However, our values are lower than those reported in two other studies [11, 78]. This could be explained by the fact that these studies measured much older pines (40 years) and native species adapted to high elevations. Another reason for the difference could be that our own allometric equation for the pine’s biomass estimation revealed a lower inclination with increasing diameter compared to the other curves (Fig. 2).

The C stocks of the natural grassland sites, except for the location of Soldados, are similar to those obtained by Bremer [74] (23.9 tC/ha) registered in tussock grass sites dominated by *Calamagrostis intermedia*, burned over 45 years ago and used for extensive Alpaca grazing as well as to those obtained by Farley et al. [11] (19.4–22.9 tC/ha) in páramo grassland burned 9 years ago with Alpaca grazing and burned 15 years ago with no grazing. We registered the highest value of C (38.2 t/ha) in NG at our highest elevation (3720 m a.s.l.). This was caused by the fact that the tussock grasses were very long and dense, in addition to the presence of tall shrubs in two study plots. Shrubs’ biomass can considerable increase aboveground carbon, as was registered in the páramos of the Yacuri National Park that is located in a southern region from our study area [79].

The carbon stock obtained in grazed sites (G) ranged from 4.7 to 9.5 tC/ha, except for the location of Nero which revealed an extremely high value of 18.3 tC/ha. These values are similar to those obtained by Hofstede et al. [80] in a study of the páramos of Colombia, in the Parque Nacional de los Nevados. In this study, Hofstede distinguished several categories of páramo: in ‘moderately grazed and burned páramo’ he registered 10.6 tC/ha, in ‘heavily grazed and burned páramo’ 4.3 tC/ha, and in páramo ‘heavily grazed without recent burning history’ 7.7 tC/ha. In another study located 150 km north-east from our study area, in the Andes of Central Ecuador, Ramsay [75] measured the biomass of a páramo grassland extensively grazed by cattle and horses, and regularly burned every 2 to 4 years. In his study Ramsay measured 4.0 to 4.2 tC/ha. These values are lower than ours, probably because Ramsay did not include litter in his measurements, which is an important component of the grasslands. Furthermore, he estimated a low net annual productivity for these páramo sites, mainly attributed to physiological water limitation.

When the elevation increases, also the physical conditions of the habitat change dramatically. These changes are collectively known as elevational gradients. They are associated with changing components such as temperature, wind velocity, atmospheric gas composition, water availability, nutrient deposition and cycling, soil weathering, and solar irradiance [81]. All of these components influence the vegetation type, composition and primary production, and through this, the input of SOC. In addition, accumulation of carbon is directly influenced by temperature, soil weathering and water availability.

Native Páramo plants are well adapted to the extreme temperatures occurring in the páramo, mainly low night temperatures followed by strong solar radiation during the day [76], while this may not be true to the introduced pines at the higher elevations. Correspondingly, in our study we observed a negative effect of increasing elevation on tree biomass of pines (above and belowground). However, our study did not examine enough environmental factors at the different elevations to conclude that elevation is the responsible factor for limiting pines growth. Nevertheless, the poor development of the pines established in the highest regions of this study is consistent with the observations made by Morris [82] in which he recognized restricted development of planted pines in areas above 3500 m a.s.l, in this region. Moreover, across the treeline ecotone, which is the zone where the plantations of the higher locations were established, stand density and tree vitality decreases with increasing elevation [83].

#### Belowground carbon

All the aforementioned studies related to carbon stock estimations included aboveground carbon pools. However, they did not consider the calculation of belowground carbon pools, probably because it is very laborious and demands much time and effort [84, 85].

In a study [77] carried out in the central zone of the Ecuadorian páramo in an elevational range between 3790 and 4100 m a.s.l, only 0.15 tC/ha of belowground biomass were registered in *P. radiata* plantations, and 0.17 tC/ha in NG. In our study, the belowground biomass carbon represents 2.7% of the total Pi carbon stock and less than 1% of the NG and G total carbon stock. Probably the values of Cargua et al. [77] are lower than those of our study most likely because they were performed in a higher elevation range. Although the belowground biomass does not contribute a significant part to the total C stock the difference of 5.4 tC/ha between the Pi and G sites and 4.2 tC/ha to the NG sites justifies the consideration of this compartment when the land uses are compared.

#### Soil organic carbon

We found that total soil organic carbon (0–45 cm depth) was high across all sites (118.6 to 373.2 tC/ha) and at five locations significant differences exist among the three land uses. Similar to previous studies [11, 15, 28], our results revealed significant lower values of total SOC (0–45 cm) for the plantations at the locations of Irquis, La Paz, Tutupali Chico and in the superficial layer of SOC (0–15 cm) of Quimsacocha compared to G and NG. In contrast, in the location of Soldados, both plantations (Pi1 and Pi2) presented significant higher values of SOC at the superficial layer. According to some studies [86, 87], afforestation is expected to increase SOC when plantations have been established on degraded or cultivated soils. In the case of the two plantations of Soldados, none of them (Pi1 and Pi2) were established on cultivated lands. In the same year that the plantations of Soldados were established, a study [88] was carried out to evaluate the state of conservation of the Ecuadorian páramos. Through transects all over the páramo, including the location of Soldados, several factors were evaluated such as: the degree of burning and grazing, other anthropic disturbances, erosion of the place, content of organic matter and biological activity in the soil. The study classified the region of Soldados as one of the most degraded páramos. This would explain the accumulation of SOC caused by the plantations of Soldados. Although the intention of these plantations was not to recover the soils, they obviously fulfilled this function. Nevertheless, as has been shown in a study in the Peruvian Andes [89], native species can be more successful in regenerating degraded soils.

The differences in SOC values that occur within each elevation range, and within some sites, highlight the heterogeneity that can exist between the categories of land use. In Hofstede’s study [25], in which the impact of pine plantations on soil carbon along the Ecuadorian páramo was studied, it was concluded that it is difficult to generalize the effects of the plantations since they vary based on environmental factors, land use history, and management of the plantations [25]. In addition to the aforementioned factors, other researcher suggest that the effects are also be influenced by edaphic and pedogenic factors [16].

## Conclusion

This study indicates that afforestation with *P. patula* in the páramo has enhanced biomass carbon stocks in comparison with natural grassland and grazed páramo. The plantations stored more biomass aboveground, mainly in the sites located in the lower elevational areas. In the higher areas, however, the pines’ biomass production was very limited. Furthermore, as in other studies [11, 15, 28] in most of the locations we registered a loss of soil carbon, as well as compaction and acidification of the soil. Therefore, afforestation in the highest zones of the páramo for the purpose of CO_2_ mitigation is not an advisable option.

Besides the tradeoff of belowground for aboveground carbon, the carbon stored in the pines is more vulnerable to be released to the atmosphere caused by fire in comparison to the carbon stored in the soil, which is a more stable pool over a longer period of time. Even though most of the carbon afforested stocks were higher, those of natural grassland were also high, which confirms that native grasslands can be an effective carbon store as well [15, 90, 91]. This study suggests that forestry plans should be assessed case by case, considering not only the limiting factor of elevation, but also the site quality especially affected by the type of previous land use. It is important to consider that the overall assessment of carbon sequestration projects depends not only on the development of the trees but also on the socioeconomic factors. If the demands and the local timber market are not considered, these projects create false expectations and disappointment on the part of the landowners [34]. Furthermore, these land use changes compromise other páramo ecosystem services such as water regulation and supply and biodiversity conservation which are factors that should be included when assessing the feasibility of these projects.

## Data Availability

The data supporting our conclusions are available either in the paper itself or in the links listed in the references. Additional data may be requested from the corresponding author on reasonable request.

## Abbreviations

ABU: Adult bovine unit
AG: Aboveground
BD: Bulk density
BG: Belowground
C: Carbon
CDM: Clean Development Mechanism
DBH: Diameter at breast height
Eq: Equation
FSS: Fine soil stock
G: Grazed páramo
IPCC: Intergovernmental Panel on Climate Change
NG: Páramo natural grassland
Pi: Pine plantation site
SOC: Soil organic carbon
UNFCCC: United Nations Framework Convention on Climate Change
